# Cross-talk between human airway epithelial cells and 3T3-J2 feeder cells involves partial activation of human MET by murine HGF

**DOI:** 10.1371/journal.pone.0197129

**Published:** 2018-05-17

**Authors:** Robert E. Hynds, Kate H. C. Gowers, Ersilia Nigro, Colin R. Butler, Paola Bonfanti, Adam Giangreco, Cecilia M. Prêle, Sam M. Janes

**Affiliations:** 1 Lungs for Living Research Centre, UCL Respiratory, University College London, London, United Kingdom; 2 CRUK Lung Cancer Centre of Excellence, UCL Cancer Institute, University College London, London, United Kingdom; 3 The Francis Crick Institute, London, United Kingdom; 4 Dipartimento di Scienze Cardio-Toraciche e Respiratorie, Universita’ degli Studi della Campania “L. Vanvitelli”, Naples, Italy; 5 Stem Cell and Regenerative Medicine Section, UCL Institute of Child Health and Great Ormond Street Hospital, London, United Kingdom; 6 Institute of Immunity and Transplantation, University College London, London, United Kingdom; 7 Centre for Cell Therapy and Regenerative Medicine, School of Biomedical Sciences, The University of Western Australia, Perth, Australia; 8 Institute for Respiratory Health, University of Western Australia, Perth, Australia; University of Alabama at Birmingham, UNITED STATES

## Abstract

There is considerable interest in the *ex vivo* propagation of primary human basal epithelial stem/progenitor cells with a view to their use in drug development, toxicity testing and regenerative medicine. These cells can be expanded in co-culture with mitotically inactivated 3T3-J2 murine embryonic feeder cells but, similar to other epithelial cell culture systems employing 3T3-J2 cells, the aspects of cross-talk between 3T3-J2 cells and human airway basal cells that are critical for their expansion remain largely unknown. In this study, we investigated secreted growth factors that are produced by 3T3-J2 cells and act upon primary human airway basal cells. We found robust production of hepatocyte growth factor (HGF) from fibroblast feeder cells following mitotic inactivation. Consistent with the limited cross-species reactivity of murine HGF on the human HGF receptor (MET; HGFR), MET inhibition did not affect proliferative responses in human airway basal cells and HGF could not replace feeder cells in this culture system. However, we found that murine HGF is not completely inactive on human airway epithelial cells or cancer cell lines but stimulates the phosphorylation of GRB2-associated-binding protein 2 (GAB2) and signal transducer and activator of transcription 6 (STAT6). Although HGF induces phosphorylation of STAT6 tyrosine 641 (Y641), there is no subsequent STAT6 nuclear translocation or STAT6-driven transcriptional response. Overall, these findings highlight the relevance of cross-species protein interactions between murine feeder cells and human epithelial cells in 3T3-J2 co-culture and demonstrate that STAT6 phosphorylation occurs in response to MET activation in epithelial cells. However, STAT6 nuclear translocation does not occur in response to HGF, precluding the transcriptional activity of STAT6.

## Introduction

The murine trachea and proximal human airways are lined by a pseudostratified epithelium consisting of basal epithelial stem/progenitor cells and differentiated mucosecretory and multiciliated cells [1–3]. Human airway epithelial cells can be expanded *in vitro* for a limited number of population doublings using serum-free bronchial epithelial growth medium (BEGM) [4]. We and others have shown that a technique first used to expand primary epidermal keratinocyte cell culture improves the longevity of airway basal cell cultures and allows prolonged retention of airway epithelial differentiation capacity in culture [5–7]. In this culture system, epithelial cells are co-cultured with 3T3-J2 mouse embryonic feeder cells in the presence of a RHO-associated protein kinase (ROCK) inhibitor, Y-27632 [8–11]. Despite the remarkable rate of proliferation of primary cells induced by these conditions, basal cells remain karyotypically stable, express markers typical of adult airway basal stem/progenitor cells and do not express those characteristic of embryonic or induced pluripotent stem cells [5]. The availability of a system for extensive expansion of primary human airway epithelial cells offers the opportunity for more realistic *in vitro* airway models, higher throughput airway drug and toxicity testing [12] and may have applications in regenerative medicine [13]. However, despite many decades of research in which keratinocytes have been co-cultured with 3T3-J2 feeder cells and the application of these cells in epidermal and corneal cell therapies [14–19], the signalling processes involved in the cross-talk between these cell types in co-culture are only partially understood [20].

In this study, we set out to characterise the nature of 3T3-J2 feeder cell cross-talk with human airway basal cells. Using phospho-receptor tyrosine kinase arrays, we identify murine hepatocyte growth factor (HGF) as a feeder cell factor capable of phosphorylating the HGF receptor, MET, on human airway basal epithelial cells. *In vivo*, HGF is predominantly produced by immune and stromal cell types and the MET receptor is expressed by epithelial cells, allowing mesenchymal-epithelial cross-talk. Previous *in vitro* studies indicate that HGF modulates the survival [21, 22], proliferation [23, 24], motility [25] and differentiation [26, 27] of lung epithelial cells. However, our result was surprising as mouse HGF is not thought to activate the human MET receptor [28–30]. Investigation of the downstream effects of HGF-MET signalling in 3T3-J2-basal cell co-culture confirmed that feeder cell-derived HGF does not affect human airway basal cell proliferation and so cannot underlie the trophic effects of 3T3-J2 cells in this system. Interestingly, HGF-MET signalling did lead to phosphorylation of the established MET target GAB2 and a previously undescribed MET target, STAT6, so murine HGF is not entirely inactive on human airway epithelial cells but rather activates only a subset of targets downstream of MET. HGF-induced phosphorylated STAT6 is retained in the cytoplasm and as such does not activate STAT6-dependent transcriptional programmes.

## Materials and methods

### Cell culture

Ethical approval for the isolation of human airway tracheobronchial epithelial cells from patient biopsies was obtained through the National Research Ethics Committee (REC references 06/Q0505/12 and 11/LO/1522) and patients gave informed, written consent. Primary human airway basal cells were obtained from adult endobronchial biopsies taken from regions of healthy airway mucosa and expanded either in bronchial epithelial growth medium (BEGM; Lonza) or in co-culture with mitotically inactivated 3T3-J2 fibroblast feeder cells in epithelial cell culture medium containing epidermal growth factor (EGF) and Y-27632 (3T3+Y) as previously described [5].

3T3-J2 fibroblast feeder cells were expanded in DMEM (Invitrogen) containing 1X penicillin/streptomycin (Gibco) and 9% bovine serum (Invitrogen). 3T3-J2-conditioned medium was prepared by inactivating 3T3-J2 fibroblasts with 0.4 μg/ml mitomycin C (Sigma-Aldrich) for 2 hours and creating feeder layers at a 20,000–30,000 cells/cm^2^ density [31]. 24 hours after seeding, fibroblast growth medium was removed and replaced with epithelial cell culture medium without Y-27632 for 24 hours. This was repeated a maximum of two times; that is, medium was collected 48 and 72 hours after cell seeding. 48-hour medium was stored at 4°C until 72-hour medium was collected and these were combined, filtered using a 0.45 μm filter and frozen at -80°C until use. Y-27632 was added immediately before use where indicated.

### Antibody arrays

Proteome profiler human phospho-receptor tyrosine kinase (RTK) antibody arrays (R&D Systems) were performed according to the manufacturer’s instructions. 500 μg fresh protein lysates were extracted from human airway epithelial cells grown in bronchial epithelial growth medium (BEGM; Lonza) and treated with media as indicated for 30 minutes. Lysates were incubated with pre-blocked nitrocellulose membranes overnight at 4°C on a rocking platform. Phosphorylated RTKs were detected using Luminata Crescendo HRP substrate (Merck Millipore) and imaged by X-ray film exposure.

### Western blotting

Cell lysis was performed using RIPA buffer containing 1X Halt protease and phosphatase inhibitor cocktail (Thermo Fisher). After scraping, cell lysates were transferred to microfuge tubes, incubated at 4°C on a rotating wheel for 30 minutes and centrifuged at 14,000 x *g* for 10 minutes before supernatants were transferred to a clean microfuge tube. After quantification by BCA assay, 20 μg protein was denatured by heating at 95°C for 10 minutes in Laemmli sample buffer (Sigma-Aldrich), separated on 4–12% Bis-Tris gels (Invitrogen) and transferred onto nitrocellulose membranes using the iBlot system (Invitrogen). Blots were blocked with tris-buffered saline containing 0.1% Tween-20 (TBST; Sigma-Aldrich) and 5% skimmed milk powder (Sigma-Aldrich) for 1 hour at room temperature on a roller mixer. Blots were incubated with primary antibodies [MET: #3135, #3077, #3133 and #8198; GAB2: #3882 and #3239; STAT6: #9361, #5297 and GTX113273 (GeneTex); α-Tubulin: #9099 (Cell Signaling Technology unless otherwise stated)] in either TBST containing 5% BSA or TBST containing 5% skimmed milk powder on a roller mixer at 4°C overnight. After washing a further 3 times with TBST for 3 minutes, blots were incubated with species-appropriate HRP-conjugated secondary antibodies (Cell Signaling Technology) for 1 hour at room temperature on a roller mixer. After washing 3 times with TBST for 3 minutes, blots were developed using Luminata Crescendo HRP substrate (Merck Millipore) and imaged using an ImageQuant LAS 4000 system (GE Healthcare). For re-probing, blots were washed briefly with TBS and incubated with Restore PLUS western blot stripping buffer (Thermo Fisher) for 15 minutes at room temperature on a roller mixer.

For experiments involving subcellular fractionation, fractions were isolated using a subcellular protein fractionation kit (Thermo Fisher) according to the manufacturer’s instructions. Resulting lysates were BCA assayed to normalize protein concentration and western blotted as described above.

### siRNA-mediated knockdown

GAB2 and non-silencing Silencer Select siRNAs were purchased from Life Technologies. Transfection was performed in primary human airway epithelial cells grown in BEGM (Lonza) using Interferin (Polyplus Transfection) according to the manufacturer’s instructions. Where indicated, cells were stimulated with vehicle control or recombinant human hepatocyte growth factor (hHGF) for 30 minutes after 72 hours of knockdown and analysed by western blotting as described above.

### Co-immunoprecipitation (IP)

A431 cells were grown in 10 cm dishes until 80% confluence. After overnight serum starvation in DMEM (Gibco), cells were treated with either a vehicle control, 3 ng/ml hHGF or 50 ng/ml hHGF (Peprotech) for 30 minutes before lysis in IP Cell Lysis Buffer (Cell Signaling). Protein concentration was normalised to 2 mg/IP in 1 ml volume and lysates were incubated with 25 μl primary antibody overnight at 4°C on a rotating wheel. Dynabeads (Protein A; Thermo Fisher) were used to isolate antibody and bound protein from the lysates according to the manufacturer’s instructions. After washing, beads were resuspended in 20 μl Laemmli sample buffer and heated to 70°C for 10 minutes. Beads were removed by centrifugation and samples were run on 4–12% Bis-Tris gels (Invitrogen). Transfer and western blotting was performed as described above.

### Enzyme-linked immunosorbent assays (ELISAs)

Secretion of HGF by 3T3-J2 cells following mitotic inactivation was assessed using a mouse HGF DuoSet ELISA kit (R&D Systems; DY2207) performed according to the manufacturer’s instructions. 3T3-J2 medium consisted of DMEM (Gibco) supplemented with 1X penicillin/streptomycin (Gibco) and 9% bovine serum (Gibco). Cells were trypsinised and seeded in fresh medium at feeder density following 2 hours of incubation with 0.4 μg/ml mitomycin C (Sigma-Aldrich) and medium was collected for analysis and refreshed after 24 hours, 48 hours and 72 hours.

### Proliferation assays

To analyse EdU uptake, primary human airway epithelial cells were cultured in 3T3+Y until approximately 70% confluence when they were washed with PBS and feeder cells were removed by taking advantage of their greater trypsin sensitivity. After washing in DMEM containing 10% FBS and then PBS, epithelial cells were treated with media as indicated for 12 hours. Cultures were treated with 10 μM EdU (Life Technologies Click-iT EdU Alexa Fluor 488) for the final 1 hour of the experiment. Single cell suspensions were obtained by trypsinisation, cells were stained according to the manufacturer’s instructions and then co-stained with DAPI. Flow cytometry was performed using an LSRFortessa (BD Biosciences) and analysed using FlowJo 10.0.6 (TreeStar).

For cell counting assays, primary human airway epithelial cells previously grown in 3T3+Y were seeded in T25 flasks at a density of 5 x 10^5^ cells in the following conditions: 3T3 co-culture in medium containing 5 μM Y-27632 and vehicle protein (3T3+Y+Vehicle), with medium alone (i.e., no feeder cells; Y+Vehicle), or medium containing Y-2632 and either 3 ng/ml or 50 ng/ml hHGF. After 24, 48 and 72 hours, cells were trypsinised and counted. After 72 hours, cells in a fourth condition were re-seeded in the same conditions in T25 flasks at a density of 5 x 10^5^ cells per flask and counted after an additional 96 hours of culture.

For MTT assays, primary human airway epithelial cells previously grown in epithelial cell culture medium without Y-27632 [DMEM/F12 in a 3:1 ratio, 1X penicillin–streptomycin, 10% fetal bovine serum, 1% adenine, hydrocortisone (0.4 μg/ml), EGF (10 ng/ml), insulin (5 μg/ml), 0.1 nM cholera toxin, 2 x 10^−9^ M T3 and gentamicin (10 μg/ml)] were seeded in 96-well plates at a density of 5,000 cells/well for 24 hours without feeder cells or EGF. The following day, cells were treated in this medium with additional growth factors as indicated. Cells were cultured for 10 days and one medium change was performed on day 5 of culture. At the given end-points, adherent cells were stained with MTT dye solution (10 μl of 1:10 diluted MTT stock solution in culture medium) for 3 hours at 37°C. After incubation, the medium was removed and 100 μl dimethyl sulfoxide (DMSO; Sigma-Aldrich) was added to dissolve the MTT crystals. The eluted specific stain was measured using a spectrophotometer (560 nm).

### Colony formation assays

For colony-forming assays in 3T3+Y, 1000 cultured airway epithelial cells previously grown in 3T3+Y were seeded per well of a 6-well plate containing inactivated 3T3-J2 feeder cells in epithelial culture medium with Y-27632 and either a vehicle control or 100 nM PF-04217903, a small molecule MET inhibitor. Cultures were maintained for 10 days and medium was changed every other day. Colonies were stained using crystal violet (Sigma-Aldrich) at room temperature for 20 minutes and washed repeatedly in water. Colonies of more than 10 cells were counted manually using a light microscope.

For colony-forming assays in the absence of Y-27632, 1000 cultured airway epithelial cells previously grown in epithelial cell culture medium without Y-27632 [DMEM/F12 in a 3:1 ratio, 1X penicillin–streptomycin, 10% fetal bovine serum, 1% adenine, hydrocortisone (0.4 μg/ml), EGF (10 ng/ml), insulin (5 μg/ml), 0.1 nM cholera toxin, 2 x 10^−9^ M T3 and gentamicin (10 μg/ml)] were seeded per well of a 6-well plate containing inactivated 3T3-J2 feeder cells in the same medium without EGF. After 48 hours, medium was refreshed with medium containing growth factors as indicated. Cultures were maintained for a further 8 days and medium was changed on day 6 of culture. Colonies were stained using crystal violet (Sigma-Aldrich) at room temperature for 20 minutes and washed repeatedly in water. Colonies of more than 10 cells were counted manually using a light microscope.

### Luciferase reporter assays

STAT6 consensus sequence reporter assays were performed by plating A431 cells in 96-well plates at a density of 20,000 cells per well. After 48 hours, cells were co-transfected with STAT6 luciferase reporter (p4xSTAT6-Luc2P was a gift from Axel Nohturfft; Addgene plasmid #35554) and renilla luciferase control (pGL4.74 [hRluc/TK]; Promega) plasmids using jetPEI (Polyplus Transfection) according to the manufacturer’s instructions. 0.225 μg STAT6 reporter and 0.025 μg renilla luciferase control were added to each well. After 24 hours, cells were washed once with PBS and serum-starved in DMEM overnight. The following day, cells were growth factor-stimulated in serum-free DMEM as indicated. To quantify the luciferase activity, a dual luciferase reporter kit (Promega) was used according to the manufacturer’s instructions. Assay reagents were injected and bioluminescence was recorded using a TROPIX TR717 microplate luminometer (2 second delay, 10 second recording time).

### Immunofluorescence

Cells were cultured in 8-well chamber slides (Ibidi) and fixed in 4% PFA for 20 minutes at room temperature. Slides were washed with and stored in PBS until staining. Cells were permeabilised and blocked in PBS containing 10% FBS and 0.01% Triton X (block buffer) for 1 hour at room temperature. Primary antibodies–keratin 5 (KRT5; Abcam, ab17130), KRT14 (Covance, PRB-155P) and p63 (Abcam, ab53039)–were incubated overnight in block buffer without Triton X at 4°C. After three 5-minute PBS washes, species-appropriate secondary antibodies (AlexaFluor dyes; Molecular Probes) were incubated at a 1:500 dilution in block buffer without Triton X for 2 hours at room temperature. Cells were washed in PBS, counterstained using DAPI (1 μg/ml stock, 1:10,000 in PBS) and washed twice more in PBS. Images were acquired using a Zeiss LSM700 confocal microscope.

### Statistics

Statistical analysis was performed using GraphPad Prism as indicated in figure legends.

## Results

### Airway basal epithelial cell receptor tyrosine kinase activation by 3T3-J2-conditioned medium

**Fig 1:**
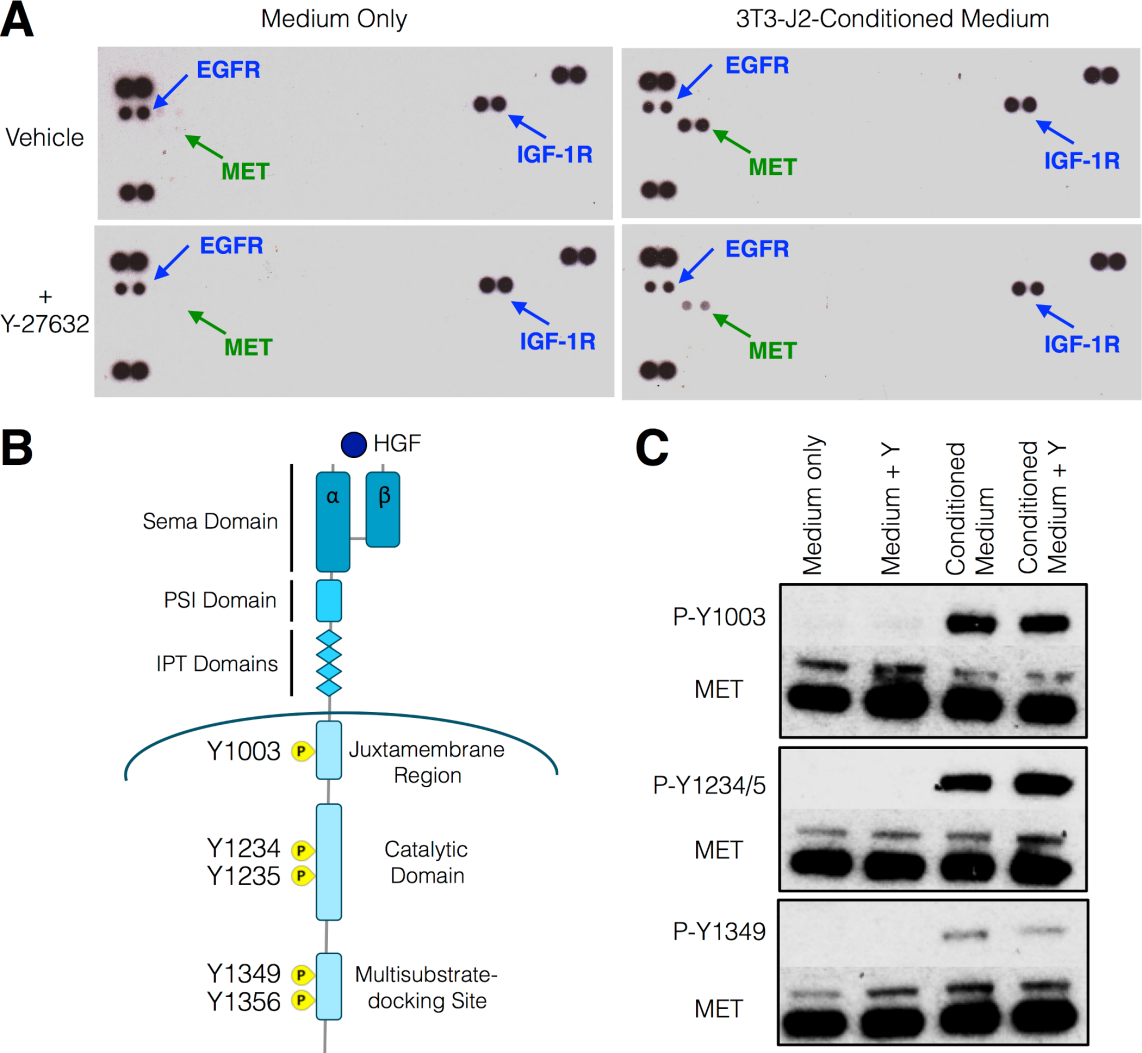
Activation of the HGF receptor (MET) in primary human airway basal epithelial cells by 3T3-J2 murine embryonic fibroblast feeder cell-conditioned medium. (A) Receptor tyrosine kinase array analysis of primary human airway basal cells stimulated for 30 minutes with 3T3-J2 feeder cell-conditioned medium. Specific phosphorylation of the hepatocyte growth factor (HGF) receptor (HGFR/MET) at Y1234/Y1235 was observed in cells stimulated with 3T3-J2-conditioned medium both in the presence and absence of Y-27632, a RHO-associated protein kinase (ROCK) inhibitor. The three duplicate spots in the top left, top right and bottom left of each array are assay reference spots. This array was performed using one donor cell culture. (B) Schematic representation of MET receptor structure showing relevant phosphorylation sites. Sema = semaphorin; PSI = plexins-semaphorins-integrins; IPT = immunoglobulins-plexins-transcription factor. (C) Western blot confirmation in independent lysates of MET phosphorylation following stimulation of primary human airway basal cells with 3T3-J2 feeder cell-conditioned medium for 30 minutes. Y indicates the presence of 5 μM Y-27632 in the culture medium. The blots shown are representative of experiments performed on three donor cell cultures.

Previous studies indicate that one or more secreted factors are responsible for the trophic effects of 3T3-J2 murine embryonic fibroblast feeder cells on human epithelial cells [32]. To investigate the nature of these signals, a receptor tyrosine kinase activation array [33] was performed on primary human airway basal epithelial cells (KRT5^+^/p63^+^; S1 Fig) stimulated with medium conditioned by 3T3-J2 fibroblasts (for a full list of phospho-receptor tyrosine kinases in array, see S1 Table). Strong activation of the epidermal growth factor receptor (EGFR) and the insulin-like growth factor 1 receptor (IGF1R) was observed in cells stimulated with both base medium and conditioned medium, consistent with the inclusion of EGF and insulin in the base medium (Fig 1A). Interestingly, the hepatocyte growth factor receptor (HGFR/MET) was strongly phosphorylated at Y1234/Y1235 by conditioned medium but not by base medium alone (Fig 1A). MET activation by 3T3-J2-conditioned medium was validated by western blot, analysing three phosphorylation sites: tyrosine 1003 (Y1003), which leads to receptor ubiquitination and recycling via endosomal pathways [34], and Y1234/Y1235, which lies within the activation loop of MET’s tyrosine kinase domain [35], were strongly phosphorylated, while Y1349, an autophosphorylation site (Fig 1B) that generates a multisubstrate-docking site [36], showed less marked phosphorylation (Fig 1C, S2 Fig and S3 Fig). Y-27632 alone was not sufficient to induce MET phosphorylation (Fig 1A and 1C) and although apparently lower MET phosphorylation in the presence of Y-27632 in the initial array (Fig 1A), this effect was not validated in subsequent western blot analyses (Fig 1C, S2 Fig and S3 Fig).

### Murine HGF activates intracellular signalling but does not affect basal cell proliferation

**Fig 2:**
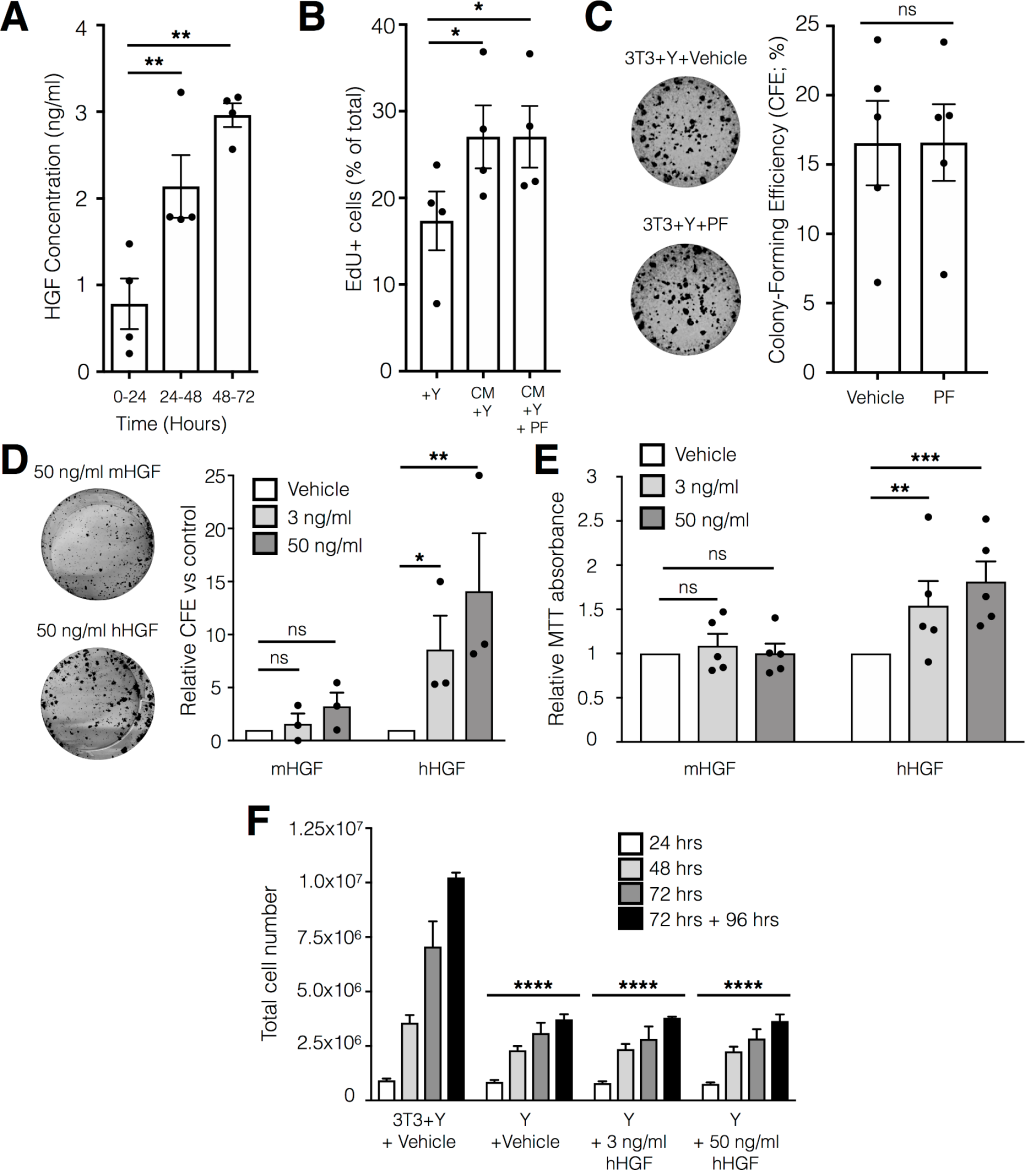
mHGF is secreted by 3T3-J2 fibroblasts following mitotic inactivation but primary human airway basal cell MET does not mediate important aspects of co-culture expansion. (A) ELISA quantification of hepatocyte growth factor (HGF) secreted into culture medium by 3T3-J2 feeder cells following mitotic inactivation with mitomycin C. Medium was collected and replaced with fresh medium after 24 and 48 hours (n = 4; mean +/- SEM; ** indicates p<0.01 using a one-way ANOVA with Holm-Sidak’s correction for multiple comparisons). (B) Flow cytometric analysis of EdU uptake in primary human airway basal cells treated with either epithelial culture medium alone (+Y), 3T3-J2-conditioned epithelial growth medium (CM+Y) or the same medium containing 100 nM PF-0421903 (CM+Y+PF), a small molecule MET inhibitor, for 12 hours (CM+Y+PF; n = 4 donor cultures performed in triplicate repeats; mean +/- SEM; * indicates p<0.05 using a one-way ANOVA with Holm-Sidak’s correction for multiple comparisons). (C) Colony-forming efficiency of primary human airway basal cells grown on 3T3-J2 feeder cells in the presence of 5 μM Y-27632 and either 100 nM PF-0421903 or a vehicle control for 10 days (n = 5 donor cultures; ns indicates not significant using a paired t test; representative wells from one donor are shown in images). (D) Colony-forming efficiency of primary human airway basal cells grown on 3T3-J2 feeder cells without EGF or Y-27632 for 10 days. 3 ng/ml or 50 ng/ml mHGF or hHGF was added as indicated (n = 3 donor cultures; * represents p<0.05 and ** represents p<0.01 using a using a two-way ANOVA with Holm-Sidak’s correction for multiple comparisons to compare each concentration of HGF to the vehicle control group; representative wells from one donor are shown in images). (E) MTT assay using primary human airway epithelial cells cultured in medium without EGF or Y-27632 for 10 days. 3 or 50 ng/ml mHGF or hHGF was added as indicated (n = 3 donor cultures; ** represents p<0.01 and *** represents p<0.001 using a using a two-way ANOVA with Holm-Sidak’s correction for multiple comparisons to compare each concentration of HGF to the vehicle control group). (F) Cell counts comparing primary human airway basal cell number in 3T3-J2 co-culture in medium containing Y-27632 and vehicle protein (3T3+Y+Vehicle) with medium alone (i.e. no feeder cells; Y+Vehicle) and medium plus either 3 ng/ml or 50 ng/ml hHGF. Cells were analysed after 24, 48 or 72 hours. In an additional condition, after 72 hours cells were passaged to the same density as at time 0 and analysed after a further 96 hours (n = 3 donor cultures performed in triplicate repeats; mean +/- SEM; **** indicates p<0.0001 using a two-way ANOVA with Holm-Sidak’s correction for multiple comparisons to compare media compositions with the 3T3+Y group).

Consistent with HGF-mediated crosstalk between fibroblast feeder cells and epithelial cells, the amount of HGF secreted into culture medium by feeder cells increased over time following mitotic inactivation (Fig 2A). However, previous work suggests that murine HGF (mHGF) does not exert biological effects on human cells as a result of a failure to initiate autophosphorylation of the multisubstrate-docking site [37]. Indeed, this site showed limited phosphorylation in airway basal cells in response to mHGF present in 3T3-J2-conditioned medium (Fig 1C, S2 Fig and S3 Fig). To investigate whether mHGF exerts biological effects on human airway basal cells, we examined their proliferation in response to 3T3-J2-conditioned medium. Basal cell proliferation increased in response to 3T3-J2-conditioned medium compared with epithelial growth medium alone as measured by EdU incorporation (Fig 2B). However, this response was not MET-dependent as the small molecule MET inhibitor PF-04217903 [38] did not reduce the amount of EdU incorporation following stimulation with 3T3+Y-conditioned medium (Fig 2B). Similarly, the presence of PF-04217903 did not affect the colony-forming efficiency of primary human airway epithelial cells (Fig 2C). Additional colony formation assays using recombinant mHGF or recombinant human HGF (hHGF) showed that, although mHGF supported the formation of some small colonies, hHGF allowed more colony formation and produced larger colonies (Fig 2D). To examine whether this was because hHGF (but not mHGF) is mitogenic for airway basal cells, we performed MTT proliferation experiments (Fig 2E), which confirmed that hHGF but not mHGF stimulated proliferation over a 10-day culture period. However, addition of hHGF to epithelial growth medium was not capable of replacing feeder cells as hHGF-treated cultures performed comparably to those in basal culture medium alone (Fig 2F).

**Fig 3:**
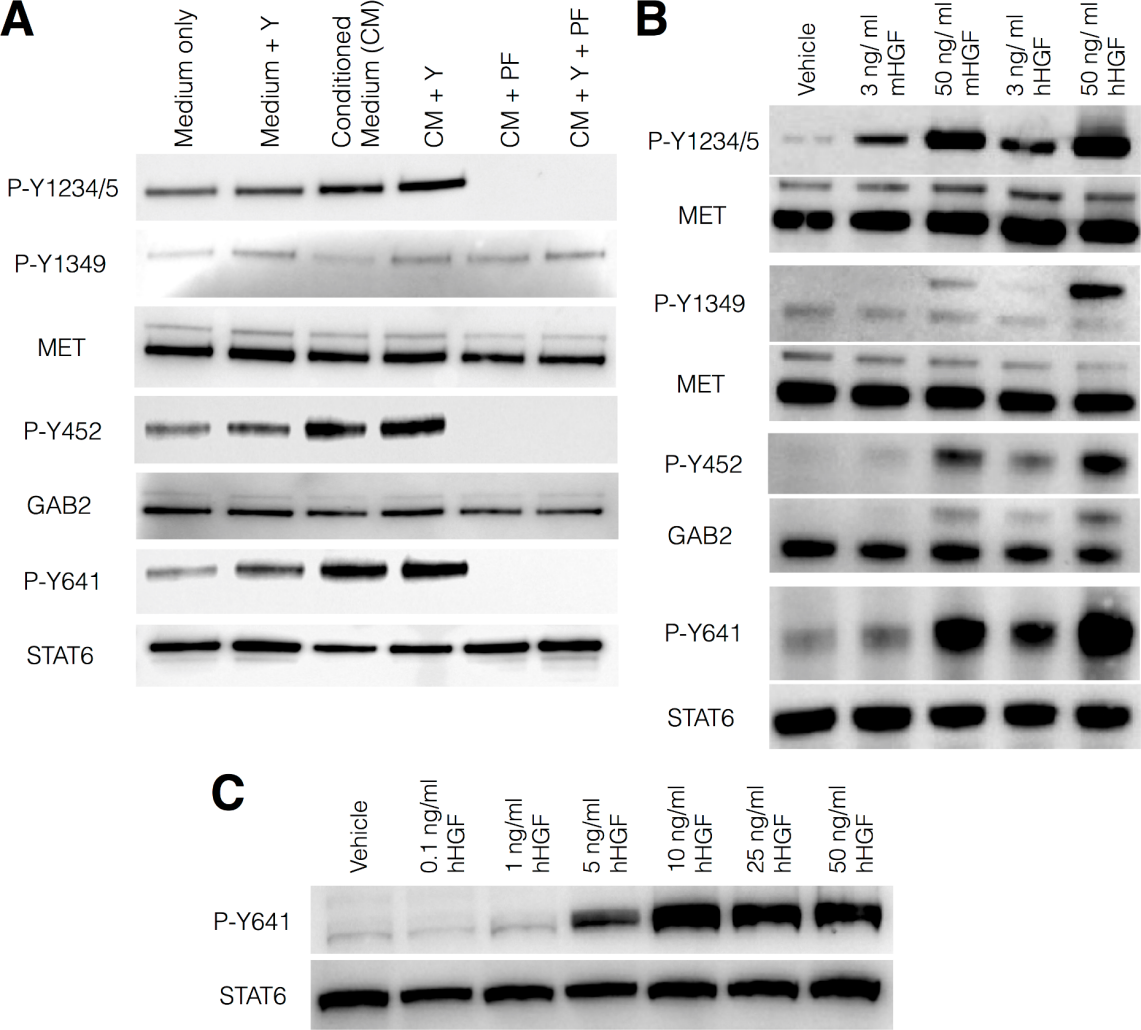
3T3-J2-conditioned medium and hHGF stimulate MET, GAB2 and STAT6 phosphorylation in primary human airway basal cells. (A) Western blot analysis of MET, GRB2-associated-binding protein 2 (GAB2) and signal transducer and activator of transcription 6 (STAT6) phosphorylation status following stimulation of primary human airway basal cells with basal or 3T3-J2-conditioned medium (CM) for 30 minutes. Y indicates the presence of 5 μM Y-27632 and PF indicates 100 nM PF-04217903. Blots are from one donor cell culture and are representative of experiments performed in three further donor cultures. (B) Western blot analysis of MET, GAB2 and STAT6 phosphorylation status in primary human airway basal cells stimulated with either 3 ng/ml or 50 ng/ml recombinant mouse (mHGF) or recombinant human hepatocyte growth factor (hHGF) for 30 minutes. Blots are from one donor cell culture and are representative of experiments performed in two further donor cultures. (C) Dose-response western blot analysis of HGF-induced STAT6 phosphorylation in primary human airway basal cells stimulated with the indicated amount of hHGF for 30 minutes. This result was replicated in a second donor cell culture.

While proliferation of airway basal cells in 3T3-J2-conditioned medium was not MET-dependent, investigation of the phosphorylation status of the MET downstream effector protein GRB2-associated-binding protein 2 (GAB2) suggested that some intracellular MET signalling events are initiated in response to mHGF (Fig 3A and S2 Fig). A previous study demonstrated that GAB2 positively influences interleukin-13 (IL-13)-dependent STAT6 phosphorylation at tyrosine 641 (Y641) in differentiated airway goblet cells [39, 40]. As such, the phosphorylation status of STAT6 in response to stimulation with 3T3-J2-conditioned medium was determined. STAT6 was robustly phosphorylated in epithelial cells treated with 3T3-J2-conditioned medium in both the absence and the presence of Y-27632, while low levels of STAT6 phosphorylation were observed in cells treated with base epithelial growth medium only (Fig 3A and S2 Fig). STAT6 phosphorylation was MET-dependent as it was prevented by treatment with PF-04217903 (Fig 3A and S2 Fig).

### Human HGF phosphorylates STAT6 in human airway basal cells

The phosphorylation events described above could be explained by the presence of co-factors in 3T3-J2-conditioned medium or by the non-physiological action of mHGF on the human MET receptor. To address this, we investigated MET, GAB2 and STAT6 phosphorylation levels following stimulation of airway basal cells with either recombinant mHGF or recombinant hHGF. In the catalytic domain (Y1234/1235), MET was phosphorylated in a dose-dependent manner in response to HGF of both species (Fig 3B and S3 Fig), while the autophosphorylated multisubstrate-docking domain of MET (Y1349), which was inefficiently activated by mHGF in 3T3-J2-conditioned medium, was only activated in response to very high concentrations of mHGF (Fig 3B and S3 Fig). Dose-response experiments revealed that hHGF treatment led to STAT6 phosphorylation in a dose-dependent manner (Fig 3C and S4 Fig). The timecourse of MET, GAB2 and STAT6 phosphorylation in response to hHGF was also comparable (S4 Fig).

**Fig 4:**
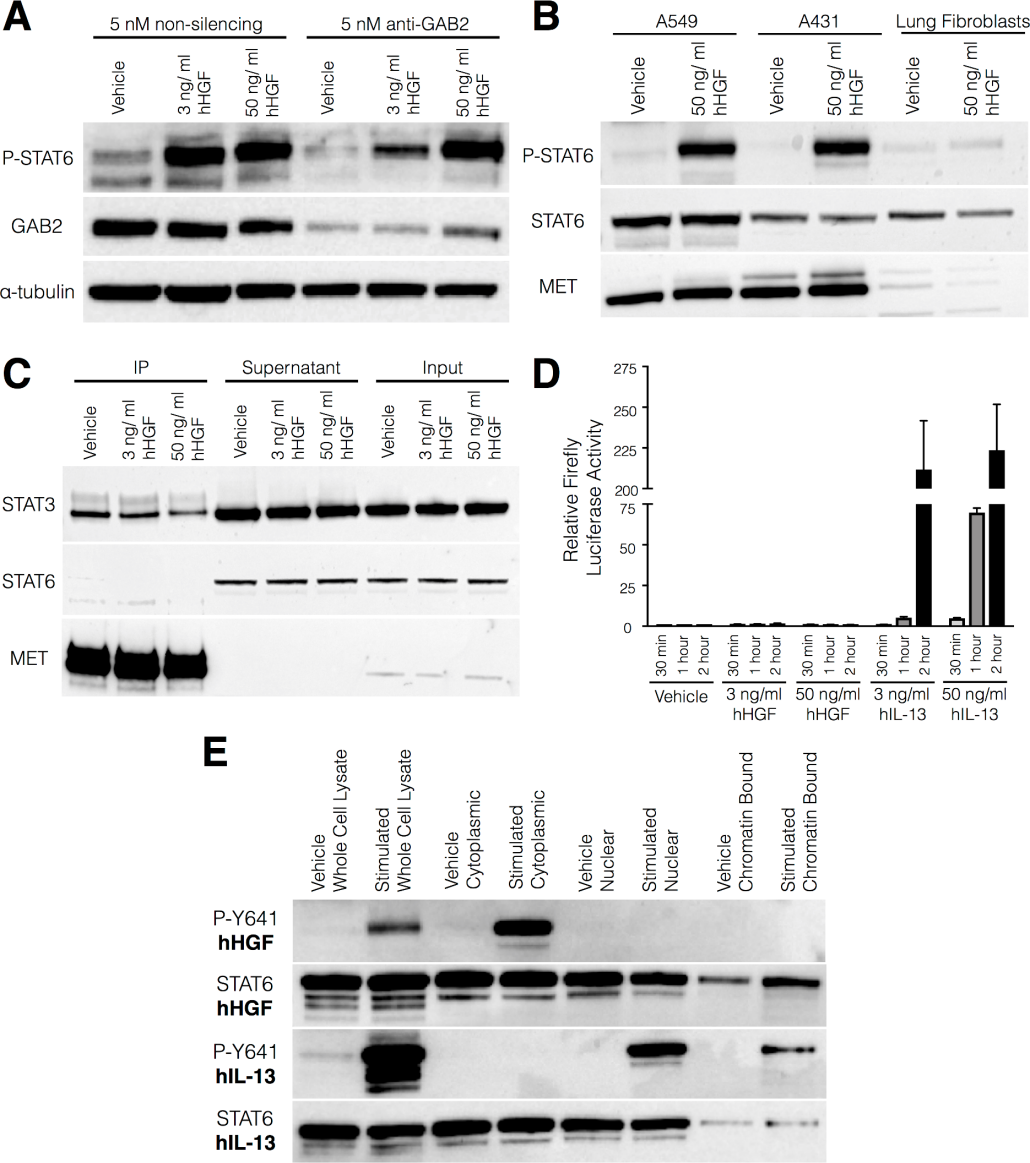
HGF-induced STAT6 phosphorylation in primary human airway basal cells occurs independently of GAB2 activity and MET receptor binding and does not result in STAT6 nuclear translocation or transcriptional activity. (A) Western blot analysis of STAT6 Y641 phosphorylation levels in human airway basal cells treated with either 5 nM non-silencing siRNA or 5 nM anti-GAB2 siRNA. Cells treated with a 0.1% bovine serum albumin (BSA) vehicle control were compared with matched cells stimulated with either 3 ng/ml or 50 ng/ml recombinant human hepatocyte growth factor (hHGF) for 30 minutes. (B) Western blot analysis of STAT6 phosphorylation in the A549 cancer cell line, the A431 cancer cell line and primary human lung fibroblasts following stimulation with either a BSA control or 50 ng/ml hHGF for 30 minutes. This blot is representative of two independent experiments. (C) Western blot analysis of co-immunoprecipitation experiment. Unstimulated A431 cancer cells were compared with matched cells stimulated with 3 or 50 ng/ml hHGF for 30 minutes. MET was immunoprecipitated using Dynabeads and the supernatant retained. STAT6 was not co-immunoprecipitated with MET but was found in the unbound protein fraction. STAT3 binding to MET was used as a positive control. These blots are representative of two experiments in A431 cells and a further experiment in primary human airway basal cells that did not detect HGF-STAT6 binding. (D) Quantification of firefly luciferase STAT6 reporter activity using luminescence in A431 cancer cells treated for 30 minutes, 1 hour or 2 hours with either a vehicle control, 50 ng/ml hHGF or 50 ng/ml recombinant human interleukin-13 (hIL-13). Values were normalised according to expression from a constitutively active renilla luciferase plasmid. No HGF-induced STAT6 activity was seen in three independent experiments. (E) Western blot analysis of STAT6 Y641 phosphorylation status in A431 cancer cells treated with 50 ng/ml hHGF or 50 ng/ml hIL-13 for 30 minutes. Whole cell lysates were obtained using RIPA buffer and compared with independent lysates prepared using a subcellular fractionation kit. These blots are representative of two independent experiments using A431 cells and two further experiments using primary human airway basal cells.

Although cooperation between IL-4/IL-13-driven STAT6 activation and MET signalling has previously been shown in myeloid cells [41], the phosphorylation of STAT6 in response to HGF has not been described so we examined STAT6 activation in this context. As GAB2 influences IL-13-induced STAT6 phosphorylation [39], we investigated whether a MET-GAB2-STAT6 pathway is activated in airway basal cells in response to HGF. However, knockdown of GAB2 using siRNA did not affect STAT6 phosphorylation (Fig 4A and S5 Fig), suggesting that HGF-induced phosphorylation of STAT6 is not dependent on GAB2.

In further mechanistic studies, we examined whether HGF-induced STAT6 phosphorylation in epithelial cancer cell lines. HGF treatment increased STAT6 Y641 phosphorylation in both A549 (lung adenocarcinoma) and A431 (epidermoid) cancer cell lines following stimulation with hHGF (Fig 4B and S6 Fig). In contrast, no upregulation of STAT6 phosphorylation was detected in HGF-treated primary human lung fibroblasts (Fig 4B and S6 Fig), consistent with the absence of MET receptor expression in these cells [42]. As STAT3 binds directly to the MET receptor via its SH2 domain [43, 44] and STAT6 also contains an SH2 domain [45], co-immunoprecipitation experiments were performed to determine whether STAT6 binds to MET following stimulation with hHGF. In immunoprecipitation assays using A431 cells, we did not detect STAT6 bound to MET, while receptor-bound STAT3 was detected (Fig 4C and S6 Fig). This finding suggests that hHGF stimulation does not lead to the direct association of STAT6 with MET and that unknown intermediate kinases are responsible for STAT6 phosphorylation in response to MET activation; however, a limitation of these experiments is that we might not have detected transient or low abundance STAT6-MET interactions.

### The HGF-induced STAT6 complex is not transcriptionally active

To investigate whether HGF induces a STAT6-dependent transcriptional response, we used a STAT6 consensus sequence luciferase reporter assay. A431 cells were transfected with a p4xSTAT6-Luc2P STAT6 luciferase reporter plasmid that contains four tandem repeats of STAT6/cEBP-binding sites (TTCN4GAA) from the human germline ε promoter sequence upstream of the luciferase gene. Cells were incubated with either a vehicle control, 3 ng/ml hHGF, 50 ng/ml hHGF, 3 ng/ml recombinant human IL-13 (hIL-13) or 50 ng/ml hIL-13, which was used as a positive control because IL-13 is known to activate STAT6-dependent transcription in epithelial cells [46, 47]. After 2 hours, hIL-13-stimulated cells showed strong expression of luciferase, indicating active STAT6 transcription, while stimulation with hHGF had no effect on luciferase expression (Fig 4D), suggesting that HGF does not activate STAT6-dependent transcription.

The lack of HGF-induced, STAT6-dependent transcriptional activity in these experiments appeared to contradict our earlier findings showing that STAT6 is robustly phosphorylated at Y641, a site whose phosphorylation is associated with STAT6 dimerisation and translocation into the nucleus [40]. To investigate the basis of this, we used subcellular fractionation to reveal the localisation of STAT6 following stimulation with either hHGF or hIL-13. After 2 hours, phosphorylated STAT6 was found in the nucleus of cells stimulated with IL-13 but remained in the cytoplasm of cells stimulated with hHGF (Fig 4E and S7 Fig), explaining the lack of STAT6 transcriptional response following stimulation with HGF.

## Discussion

3T3-J2 mouse embryonic feeder cells are supportive of human epithelial cell expansion *in vitro* [48] and soluble factors appear to mediate their effect(s) [32]. Data from our laboratory also indicated that secreted factors mediate the effect of 3T3-J2 co-culture but that they might be required in constant supply: conditioned medium is insufficient to fully recapitulate the effects of co-culture [49]. To address the contribution of growth factor signalling in 3T3-J2 fibroblast co-cultures with primary human airway basal cells, we used a receptor tyrosine kinase array to identify candidate pathways. Given the complex composition of feeder cell-conditioned medium, we anticipated that we would uncover a range of candidates but in fact only the HGF receptor, MET, was phosphorylated in airway basal cells following stimulation with 3T3-J2 fibroblast-conditioned medium. A limitation of the strategy employed here is that conditioned medium was collected from epithelial cell-naïve fibroblast feeder cells and it is possible that epithelial cell-derived signals modulate the feeder cell secretome in co-culture [50].

Nevertheless, HGF-MET signalling emerged as a promising candidate mediating feeder cell-epithelial cell cross-talk as it was secreted by feeder cells in increasing amounts following mitotic inactivation and partially activated MET on human epithelial cells. This is consistent with the physiological role of HGF-MET signalling, where mesenchyme-derived HGF signals to MET on epithelial cells to initiate diverse responses such as proliferation, migration, survival and differentiation [51]. The identification of MET activation in human epithelial cells stimulated with 3T3-J2-conditioned medium was particularly interesting as murine HGF is not thought to bind efficiently to the human MET receptor [52]. Indeed, autophosphorylation of the MET receptor multisubstrate-docking site was lower in response to mHGF in 3T3-J2-conditioned medium compared with hHGF. Since neither proliferation induced by 3T3-J2-conditioned medium nor colony formation was affected by MET inhibition and recombinant HGF could not replace the requirement for feeder cells, we conclude that HGF signalling is not responsible for the improvements in epithelial cell culture conditions conferred by 3T3 co-culture.

Despite this, characterisation of signalling downstream of MET in response to 3T3-J2-conditioned medium identified MET-dependent phosphorylation of GAB2 and the transcription factor STAT6, a novel target of MET signalling, and these findings were validated using recombinant hHGF. STAT6-mediated signalling occurs in response to IL-4 and IL-13, cytokines that are implicated in the pathogenesis of airway diseases [40, 53, 54]. IL-13 treatment of an epithelial cancer cell line demonstrated STAT6 activation and translocation to the nucleus. Although, HGF treatment significantly increased STAT6 activation as determined by the levels of STAT6 phosphorylation, HGF-induced phosphorylated STAT6 did not activate transcriptional targets as it did not translocate to the nucleus.

One possible explanation is that further post-translational modifications to STAT6, in addition to Y641 phosphorylation, are required to allow dimerisation and/or nuclear translocation. Alternatively, a cytoplasmic inhibitory mechanism may prevent these; a previous study showed that cytoplasmic phosphorylated STAT6 cannot bind to DNA *in vitro* but that detergent treatment confers on it DNA-binding ability [55]. Although the identity of this inhibitor and the mechanism of inhibition are unknown, one possibility is that a bound factor both prevents the nuclear import of STAT6 and masks the DNA-binding site. Importin-α5 binds competitively to the STAT1 DNA-binding site [56], giving biological precedent to this hypothesis.

Overall, our results suggest that mHGF cannot be considered completely inactive on human cells. Although the characteristic proliferative and migratory effects of HGF are lacking following stimulation with mHGF due to inefficient multisubstrate-docking site phosphorylation [37], some intracellular signalling–including the phosphorylation of GAB2 and STAT6 –proceeds. It is likely that the behavior of other cytoplasmic proteins and transcription factors are modified by the incomplete activation of MET by mHGF. While more research is clearly required to understand the factors that enable 3T3-J2-mediated epithelial cell expansion and stem cell retention, these findings draw attention to the possible relevance of complex cross-species protein interactions in this system.

## Supporting information

S1 Table Receptor tyrosine kinases assayed in the R&D systems human phospho-receptor tyrosine kinase (RTK) array kit (Catalogue Number ARY001B). (TIFF)

S1 Fig Immunofluorescence demonstrates expression of basal epithelial cell markers p63, keratin 5 (KRT5) and keratin 14 (KRT14) in both BEGM and 3T3+Y cell culture conditions. (TIFF)

S2 Fig Western blot analyses of MET, GAB2 and STAT6 phosphorylation status in primary human airway basal epithelial cells treated with 3T3-J2 conditioned medium. Y indicates the presence of 5 μM Y-27632; PF indicates 100 nM PF-04217903. This figure is associated with Figs 1C and 3A. Each group of blots (A, B and C) are biological replicates from independent donor cultures.
(TIFF)

S3 Fig Western blot analyses of MET, GAB2 and STAT6 phosphorylation status in primary human airway basal epithelial cells treated with recombinant murine and human HGF. Y indicates the presence of 5 μM Y-27632; PF indicates 100 nM PF-04217903. This figure is associated with Fig 3B and each group of blots (A, B, C and D) are from independent donor cultures.
(TIFF)

S4 Fig Additional western blot analyses of MET, GAB2 and STAT6 phosphorylation status in primary human airway basal epithelial cells treated with recombinant human HGF. (A) Western blot analysis of the phosphorylation status of STAT6 following treatment with a dose range of recombinant human HGF. This panel is associated with Fig 3C and was performed on an independent donor culture. (B) Timecourse of MET, GAB2 and STAT6 phosphorylation status in primary human airway epithelial cells in response to 50 ng/ml recombinant human HGF.
(TIFF)

S5 Fig Western blot analyses of STAT6 phosphorylation status in primary human airway basal epithelial cells treated with recombinant human HGF in the presence of anti-GAB2 siRNA. This figure is associated with Fig 4A and each group of blots (A and B) were performed on independent donor cultures.
(TIFF)

S6 Fig Additional western blot analyses of HGF-induced STAT6 phosphorylation in A431 cells. (A) This panel is associated with Fig 4B and was performed on independent cell lysates. (B) This panel is associated with Fig 4C and was performed on independent A431 cell lysates but using a lower (1 mg) protein input. (C) This panel is associated with Fig 4C and was performed on a primary human airway basal cell culture.
(TIFF)

S7 Fig Analysis of STAT6 cellular localisation by subcellular fractionation. This figure is associated with Fig 4E. (A) Subcellular fractionation confirmation for the experiment presented in Fig 4E using A431 cell lysates. (B) Replication of the experiment shown in Fig 4E in independent A431 cell lysates using 50 ng/ml hHGF and 50 ng/ml hIL-13. (C, D) Replication of our findings in A431 cancer cells in two independent primary human airway basal cell cultures using 50 ng/ml hHGF and 50 ng/ml hIL-13.
(TIFF)
