# Supplementary figures and images for: Cross-talk between human airway epithelial cells and 3T3-J2 feeder cells involves partial activation of human MET by murine HGF
Source: PLoS One. 2018 May 17;13(5):e0197129. doi: 10.1371/journal.pone.0197129 (PMC5957441; doi:10.1371/journal.pone.0197129)

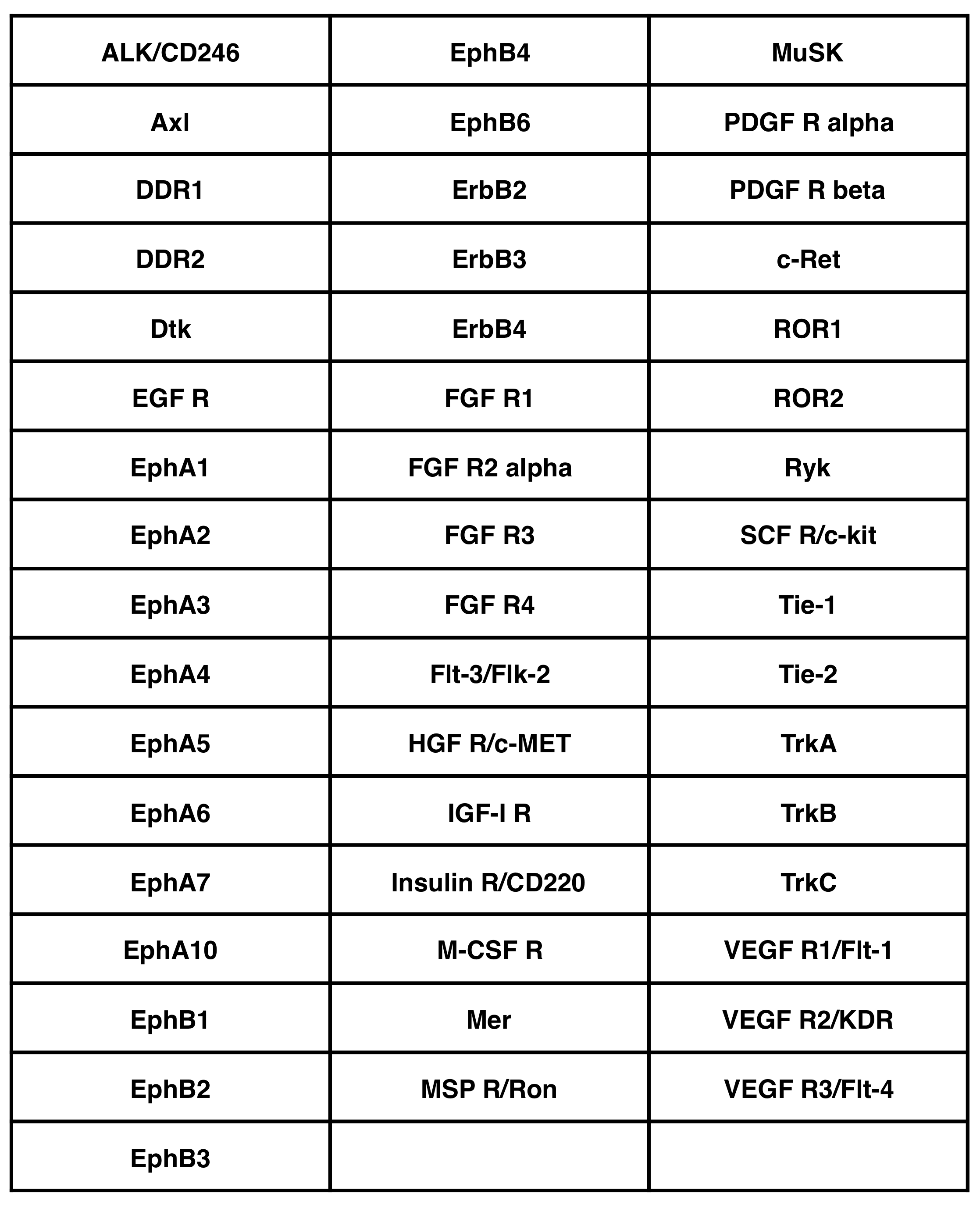

Supplement: S1 Table — (TIFF) [file pone.0197129.s001.tiff]

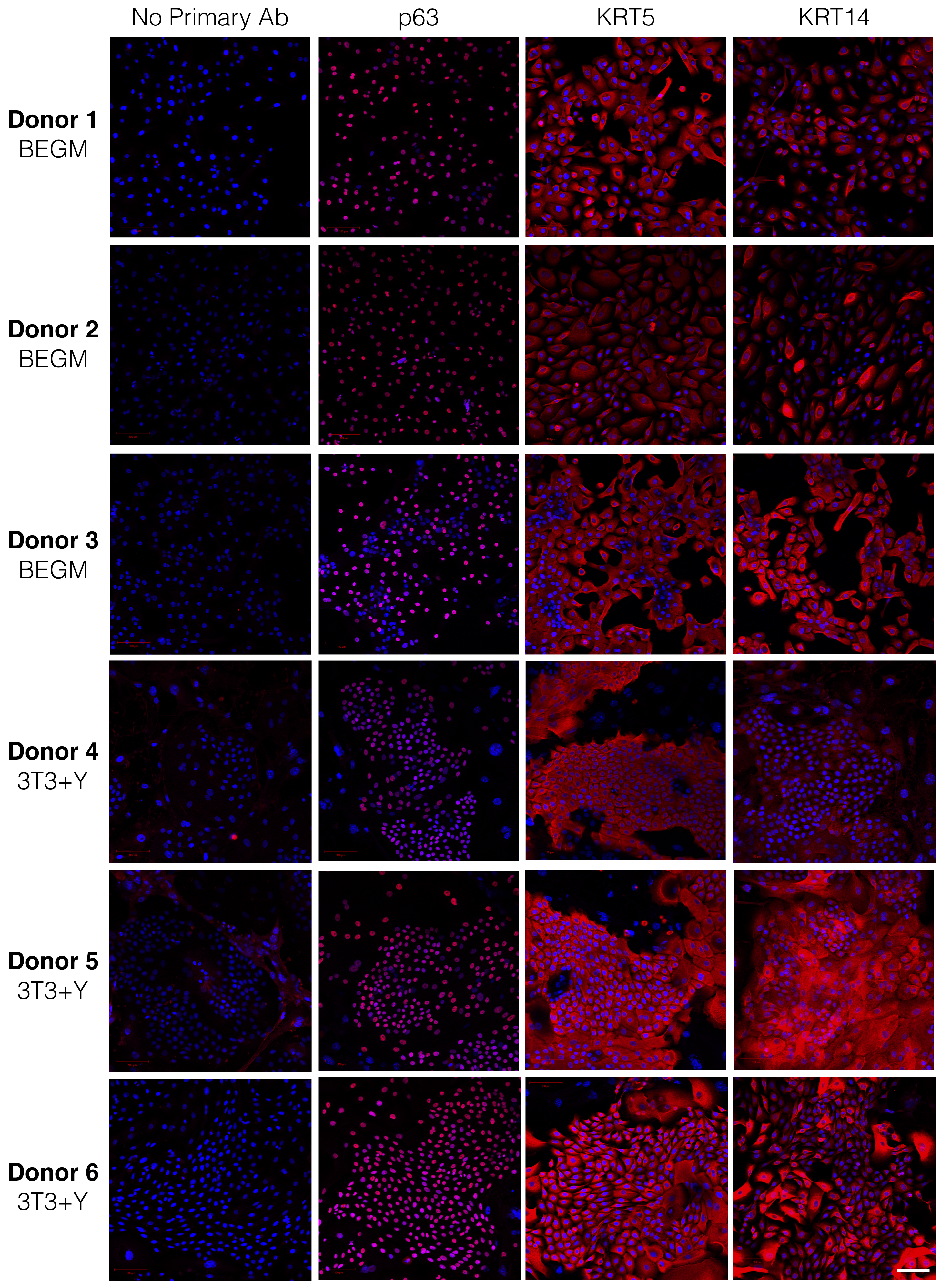

Supplement: S1 Fig — (TIFF) [file pone.0197129.s002.tiff]

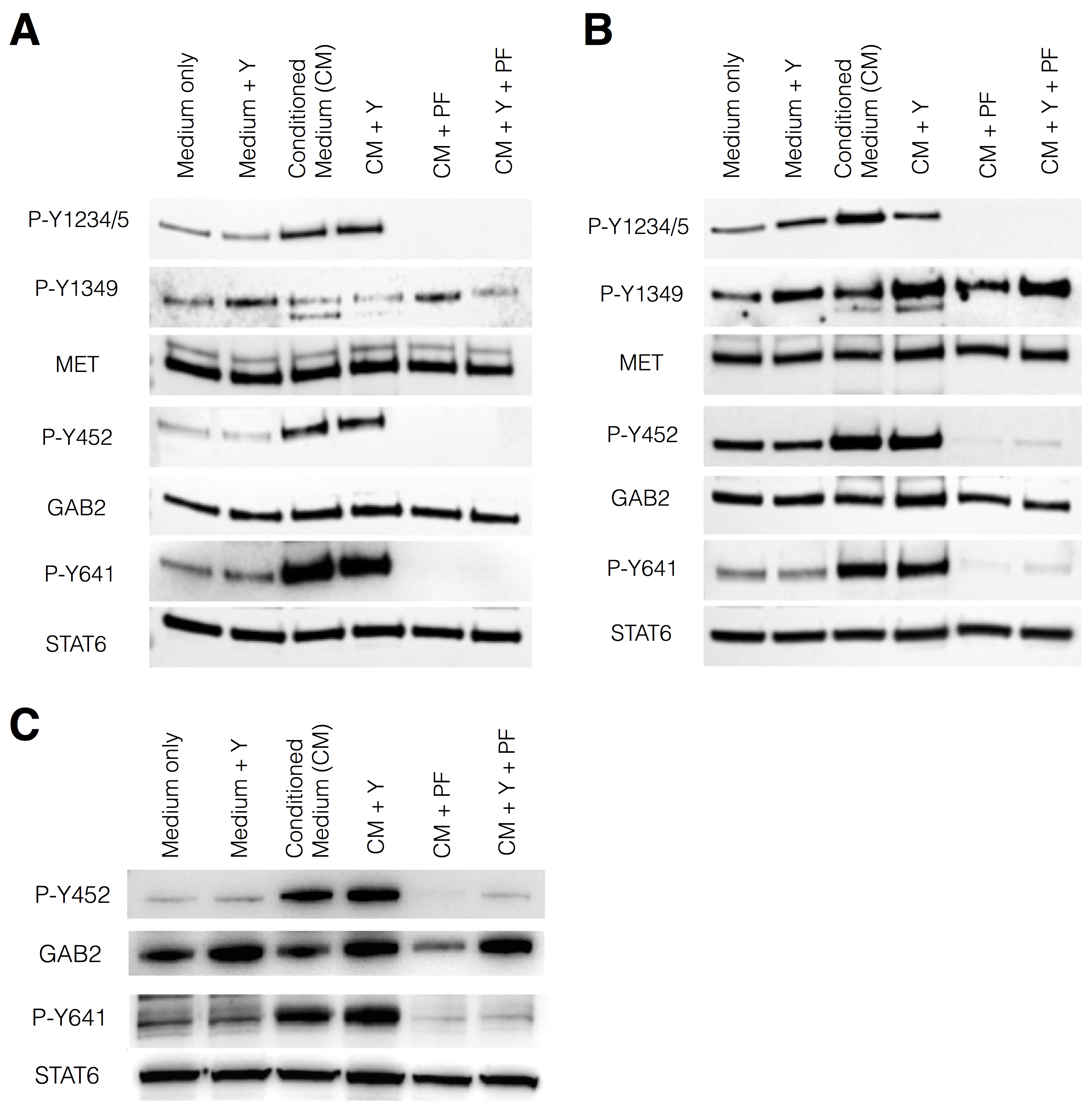

Supplement: S2 Fig — Y indicates the presence of 5 μM Y-27632; PF indicates 100 nM PF-04217903. This figure is associated with Figs 1C and 3A. Each group of blots (A, B and C) are biological replicates from independent donor cultures. (TIFF) [file pone.0197129.s003.tiff]

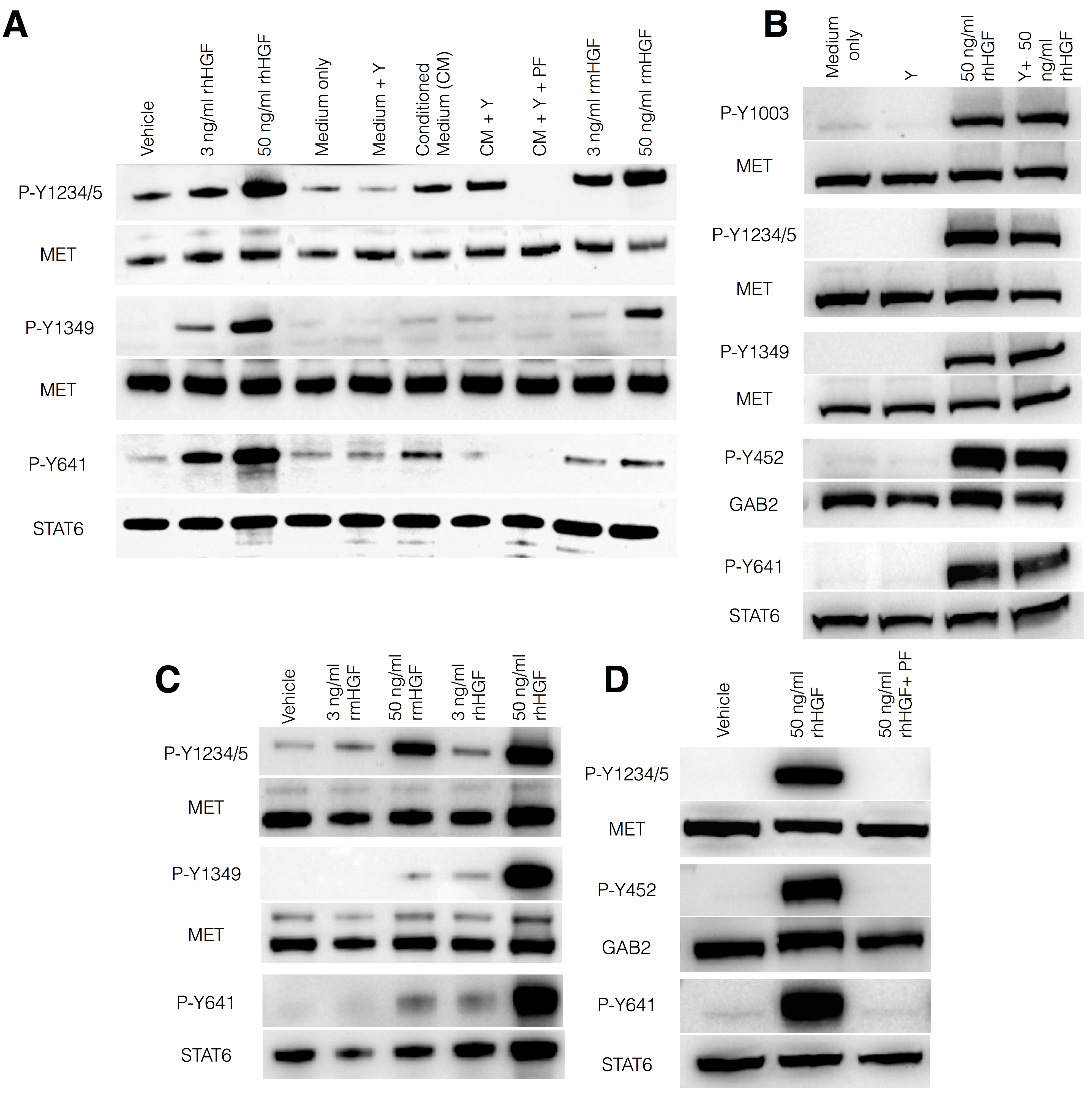

Supplement: S3 Fig — Y indicates the presence of 5 μM Y-27632; PF indicates 100 nM PF-04217903. This figure is associated with Fig 3B and each group of blots (A, B, C and D) are from independent donor cultures. (TIFF) [file pone.0197129.s004.tiff]

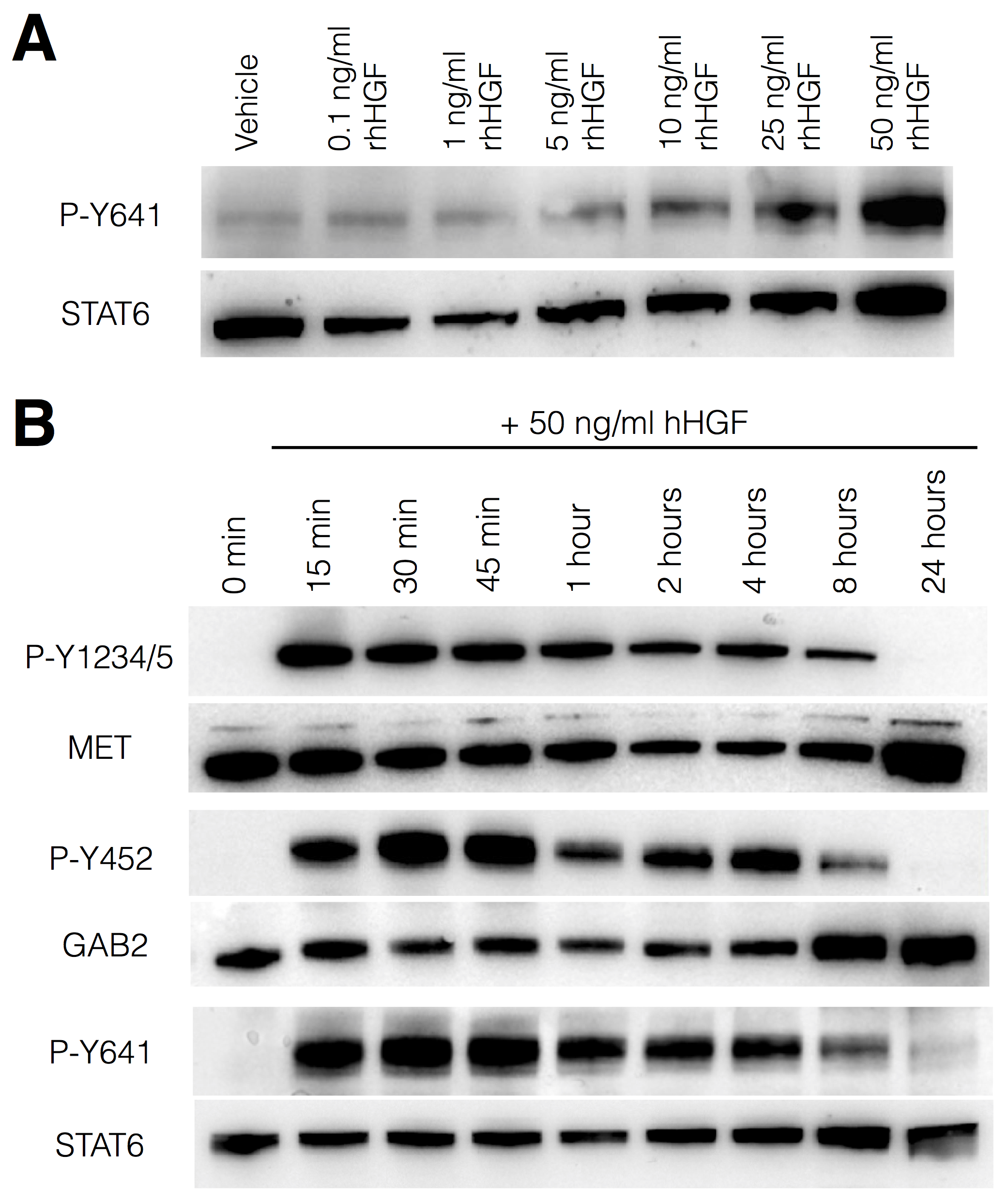

Supplement: S4 Fig — (A) Western blot analysis of the phosphorylation status of STAT6 following treatment with a dose range of recombinant human HGF. This panel is associated with Fig 3C and was performed on an independent donor culture. (B) Timecourse of MET, GAB2 and STAT6 phosphorylation status in primary human airway epithelial cells in response to 50 ng/ml recombinant human HGF. (TIFF) [file pone.0197129.s005.tiff]

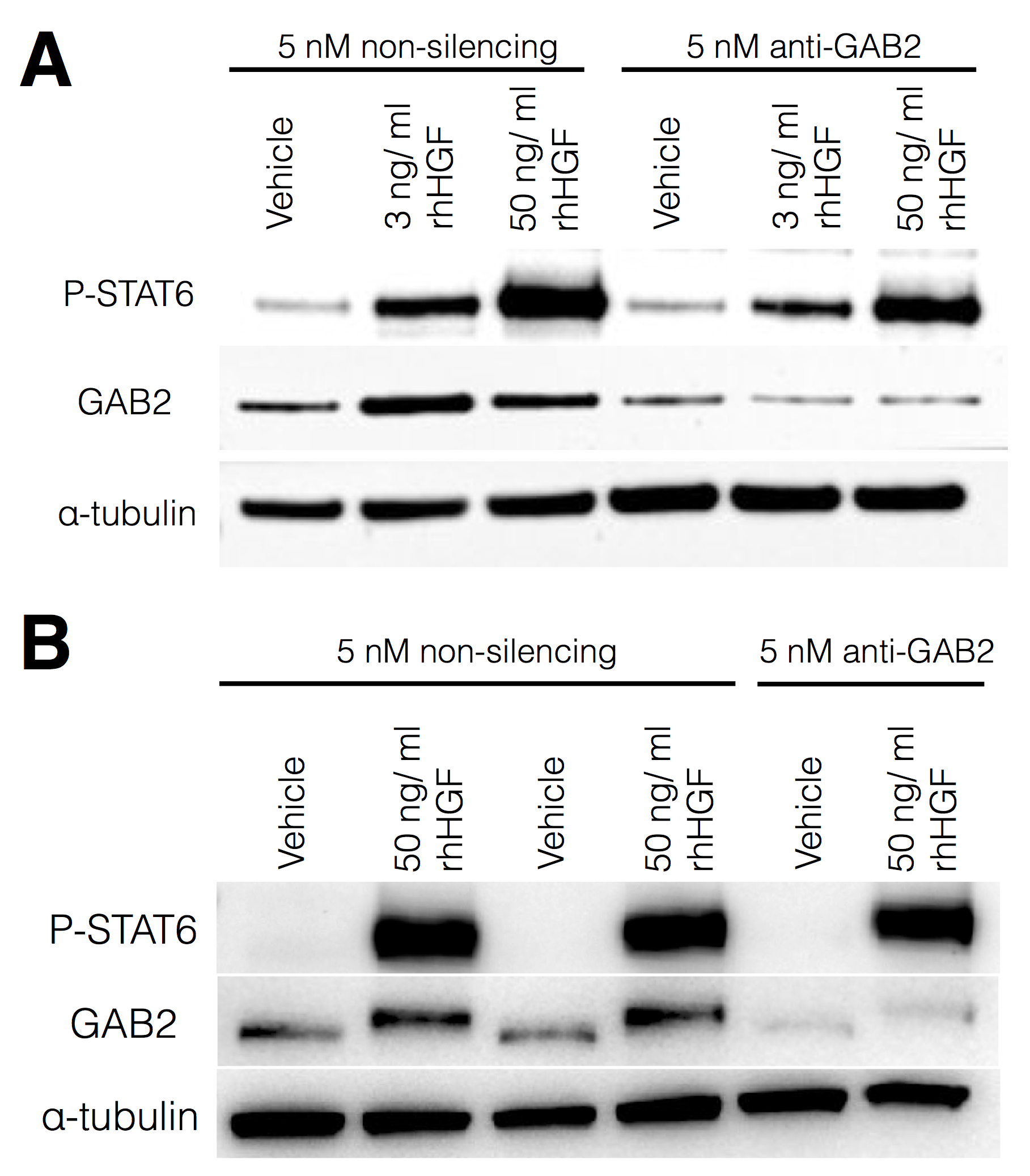

Supplement: S5 Fig — This figure is associated with Fig 4A and each group of blots (A and B) were performed on independent donor cultures. (TIFF) [file pone.0197129.s006.tiff]

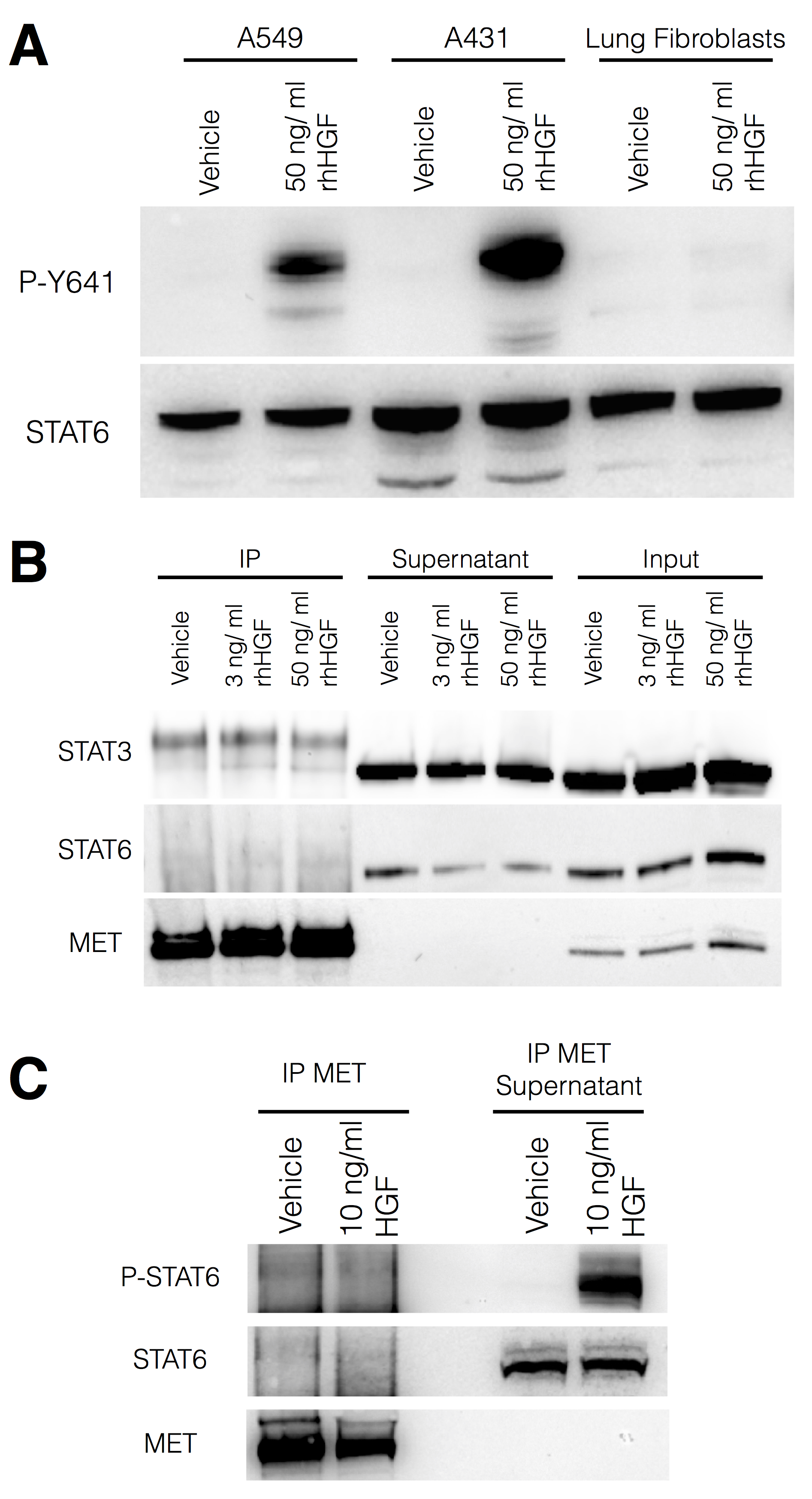

Supplement: S6 Fig — (A) This panel is associated with Fig 4B and was performed on independent cell lysates. (B) This panel is associated with Fig 4C and was performed on independent A431 cell lysates but using a lower (1 mg) protein input. (C) This panel is associated with Fig 4C and was performed on a primary human airway basal cell culture. (TIFF) [file pone.0197129.s007.tiff]

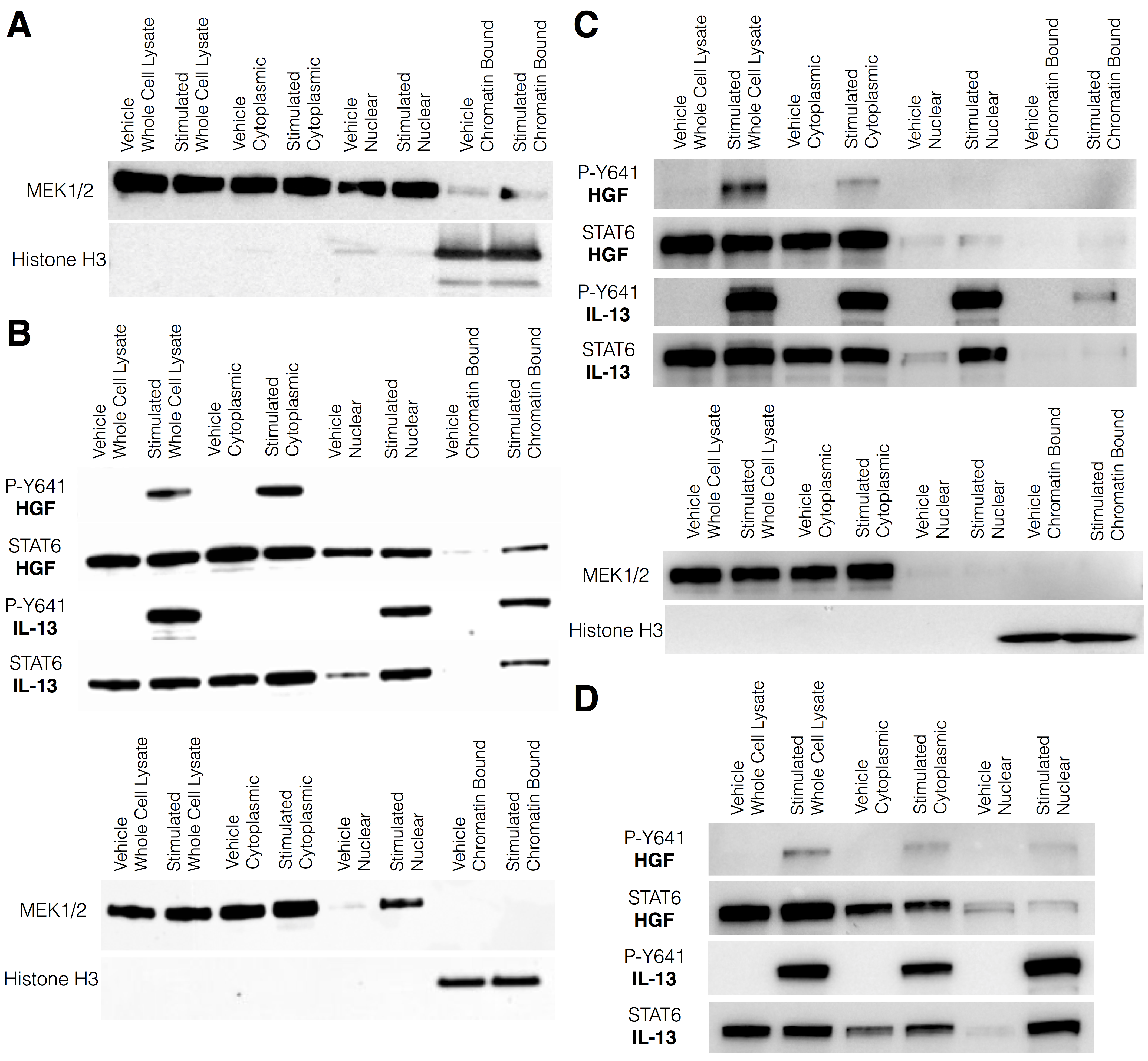

Supplement: S7 Fig — This figure is associated with Fig 4E. (A) Subcellular fractionation confirmation for the experiment presented in Fig 4E using A431 cell lysates. (B) Replication of the experiment shown in Fig 4E in independent A431 cell lysates using 50 ng/ml hHGF and 50 ng/ml hIL-13. (C, D) Replication of our findings in A431 cancer cells in two independent primary human airway basal cell cultures using 50 ng/ml hHGF and 50 ng/ml hIL-13. (TIFF) [file pone.0197129.s008.tiff]
